# Impact of Intercropping Five Medicinal Plants on Soil Nutrients, Enzyme Activity, and Microbial Community Structure in *Camellia oleifera* Plantations

**DOI:** 10.3390/microorganisms12081616

**Published:** 2024-08-08

**Authors:** Azuo Bajiu, Kai Gao, Guangyu Zeng, Yuanhao He

**Affiliations:** 1 Guangxi Key Laboratory of Special Non-Wood Forest Cultivation and Utilization, Nanning 530002, China; bjaz1999@163.com (A.B.); gaokaigxu@163.com (K.G.); 2 Key Laboratory of National Forestry and Grassland Administration on Control of Artifcial Forest Diseases and Pests in South China, Hunan Provincial Key Laboratory for Control of Forest Diseases and Pests, Key Laboratory for Non-Wood Forest Cultivation and Conservation of Ministry of Education, Key Laboratory of Forest Bio-Resources and Integrated Pest Management for Higher Education in Hunan Province, College of Forestry, Central South University of Forestry and Technology, Changsha 410004, China

**Keywords:** *C. oleifera*, medicinal plants, intercropping, soil properties, soil microorganisms

## Abstract

Intercropping medicinal plants plays an important role in agroforestry that can improve the physical, chemical, and biological fertility of soil. However, the influence of intercropping medicinal plants on the *Camellia oleifera* soil properties and bacterial communities remains elusive. In this study, five intercropping treatment groups were set as follows: *Curcuma zedoaria*/*C. oleifera* (EZ), *Curcuma longa*/*C. oleifera* (JH), *Clinacanthus nutans*/*C. oleifera* (YDC), *Fructus Galangae*/*C. oleifera* (HDK), and *Ficus simplicissima*/*C. oleifera* (WZMT). The soil chemical properties, enzyme activities, and bacterial communities were measured and analyzed to evaluate the effects of different intercropping systems. The results indicated that, compared to the *C. oleifera* monoculture group, YDC and EZ showed noticeable impacts on the soil chemical properties with a significant increase in total nitrogen (TN), nitrate nitrogen (NN), available nitrogen (AN), available phosphorus (AP), and available potassium (AK). Among them, the content of TN and AK in the rhizosphere soil of Camellia oleifera in the YDC intercropping system was the highest, which was 7.82 g/kg and 21.94 mg/kg higher than CK. Similarly, in the EZ intercropping system, the content of NN and OM in the rhizosphere soil of Camellia oleifera was the highest, which was higher than that of CK at 722.33 mg/kg and 2.36 g/kg, respectively. *Curcuma longa*/*C. oleifera* (JH) and *Clinacanthus nutans*/*C. oleifera* (YDC) had the most effect on soil enzyme activities. Furthermore, YDC extensively increased the activities of hydrogen peroxide and acid phosphatase enzymes; the increase was 2.27 mg/g and 3.21 mg/g, respectively. While JH obviously increased the urease activity, the diversity of bacterial populations in the rhizosphere soil of the intercropping plants decreased, especially the Shannon index of YDC and HDK. Compared with the monoculture group, the bacterial community abundance and structure of JH and YDC were quite different. The relative abundance of *Actinobacteriota* and *Firmicutes* was increased in YDC, and that of *Acidobacteriota* and *Myxococcota* was increased in JH. According to the redundancy analysis (RDA), pH, total potassium, and soil catalase activity were identified as the main factors influencing the microbial community structure of the intercropping systems. In conclusion, intercropping with JH and YDC increased the relative abundance of the dominant bacterial communities, improved the microbial community structure, and enhanced the soil nutrients and enzyme activities. Therefore, in the future, these two medicinal plants can be used for intercropping with *C. oleifera*.

## 1. Introduction

*C. oleifera*, an evergreen shrub also known as “Oriental Olive Oil”, is widely distributed and cultivated in central and south China [1]. It is an essential oil tree species with high nutritional value that is listed among the key healthy edible oils by the Food and Agriculture Organization (FAO) [2]. The planting area of *C. oleifera* has grown rapidly in recent years [3]; however, more and more problems have emerged, such as the waste of land resources due to the large planting gap of *C. oleifera* and low economic income in the pre-harvest period [4]. Therefore, an under-forest economy should be developed to improve the land utilization rate and increase economic benefits.

The timber-medicine compound management model is an efficient intercropping cultivation approach that can fully utilize land resources, improve soil properties and micro-ecology, and increase yields [5]. Zhang et al. [6] have shown that intercropping medicinal herbs with moso bamboo significantly increases the soil organic carbon (SOC) and AN content, while decreasing pH and AK. According to a report, intercropping *Forsythia suspensa* with *Polygonum multiflorum* has increased the yield and quality of *P. multiflorum* due to an improvement in the soil microbial ecology and quality [7]. Soil microbial diversity and richness have been identified as important factors in soil health and plant productivity [8]. Intercropping mulberry with alfalfa can increase the abundance of soil Actinomycetes and total phosphorus content, and the diversity of the dominant bacteria is related to soil pH and total phosphorus [9].

Utilizing gaps in the *C. oleifera* trees to plant green manure crops and oilseed crops instead of tending can suppress weed growth, conserve water and soil, and improve the microclimate within the forest gaps. This practice promotes the root growth and overall development of *C. oleifera* trees, aiming to achieve rapid growth and early fruiting. At present, plant intercropping with *C. oleifera* is mainly focused on annual economic crops such as *Arachis hypogaea* Linn, *Ipomoea batatas* (L) Lam, and *Glycine max*, as well as green manure crops like *Chamaecrista nictitans*, *Cassia rotundifolia Pers*, and *Lolium perenne* [2,10,11]. *C. zedoaria*, *C. longa*, *C. nutans*, *F. Galangae*, and *F. simplicissima* are common medicinal plants with high economic value, and there are no studies on their intercropping with *C. oleifera*. Therefore, we selected these five medicinal plants for intercropping with *C. oleifera* in this study, exploring the effects on the intercropping systems including soil nutrients, enzyme activities, and microbial community diversity and structure, and aiming to provide a basis for the construction of an efficient and ecological planting model of *C. oleifera*.

## 2. Materials and Methods

### 2.1. Site Description

The experimental site was located in the experimental forest farm (22°54′43″–22°56′51″ N, 108°19′08″–108°21′32″ E) of the Academy of Forestry Science of Guangxi Zhuang Autonomous Region. The region has a subtropical monsoon climate with an average annual temperature of 21.6 °C, average relative humidity of 80%, annual precipitation of 1609.1 mm, and annual frost-free period of 360 days. The soil is lateritic red soil, which is acidic.

In this experiment, seven medicinal plants were selected to intercrop with *C.oleifera*, including one-year *C. zedoaria* (EZ1),two-year *C. zedoaria* (EZ), one-year *C. longa* (JH1), two-year *C. longa* (JH), *C. nutans* (YDC), *F. Galangae* (HDK) and *F. simplicissima* (WZMT), monocropping *C. oleifera* as the control (CK). Except for the one-year *C. zedoaria* (EZ1) and two-year *C. zedoaria* (EZ), all medicinal plants were planted on 10 April 2021, without any fertilization from planting to sampling. Each medicinal plant had four replicates, resulting in a total of 32 experimental plots. Each treatment plot was 60 m × 10 m. Within a plot, four rows of the same medicinal plant were intercropped with two rows of *C.oleifera*. The spacing for EZ, JH, and YDC was 0.3 m between rows and 0.3 m between plants. WZMT had a row spacing of 0.7 m and a plant spacing of 0.5 m. HDK had a row spacing of 1 m and a plant spacing of 1 m. Plots were separated by a 2 m buffer zone.

### 2.2. Collection of Soil Samples

The samples were collected in April 2023. In each plot, soil samples were collected from three zones, such as the roots of medicinal plants (CKZ, EZZ, JHZ, HDKZ, YDCZ, and WZMTZ), the roots of *C. oleifera* (CKY, EZY, JHZ, HDKY, YDCY, and WZMTY), and the interspace between them (CKK, EZK, JHK, HDKK, YDCK, and WZMTK). In each zone, four soil samples were collected at the same distance, and after mixing, about 1 kg was taken as one sample. There were three zones in one plot, and four replicates were set. A total of 96 soil samples were collected. Soil samples were collected at a depth of 5 cm after the humus layer was removed. The soil sample was sieved to remove roots and stones (>4 mm), mixed evenly, and stored in dry ice. The samples were then transported to the laboratory and stored in a freezer at −80 °C for soil microbial diversity and structure analysis. The remaining soil samples were placed in bags, transported to the laboratory, air-dried, screened with a 100-mesh (diameter 0.150 mm) sieve, and then used in the analysis of the chemical properties and soil enzyme activities.

### 2.3. Soil Chemical Property Analyses

The soil chemical properties were determined according to the method of Bao [12]. The pH determination was conducted using the acidity titration method. The soil total organic matter (OM) was determined with the potassium dichromate method; total nitrogen (TN) was analyzed by the semi-micro-Kjeldahl method; concentrations of soil ammonium nitrogen (AN) and nitrate nitrogen (NN) were determined by UV spectrophotometry; total phosphorus (TP) was determined by the Mo-Sb colorimetric method; total potassium (TK) was determined using the NaOH melt flamer method; available P (AP) was measured by the hydrochloric acid-sulfuric acid extraction method; and available K (AK) was measured by the NH_4_OAc method. All determinations were made in triplicates.

### 2.4. Soil Enzyme Activity Analyses

Soil enzyme activity was determined according to the method of Guan [13]. Soil enzyme activities were assayed as follows: Urease activity (S-UE) was analyzed by the phenol-sodium hypochlorite colorimetric method; sucrase activity (S-SC) was measured with the 3,5-dinitrosalicylic acid colorimetric method; soil catalase (S-CAT) activity was determined by the potassium permanganate titration method; and soil acid phosphatase activity (S-ACP) was determined by the disodium phenyl phosphate colorimetric method.

### 2.5. DNA Extraction, PCR Amplification and High-Throughput Sequencing

The FastDNA Spin kit (Omega Bio-tek, Norcross, GA, USA) was used for soil DNA extraction. The extracted DNA was purified with the TIANquick Midi purification kit (Tiangen Biotechnology Co., Ltd., Beijing, China). DNA was quantified using an ND 1000 spectrophotometer (NanoDrop, Thermo Scientific, Wilmington, NC, USA) and adjusted to a final concentration of 2.5 ng/μL. DNA integrity was verified by gel electrophoresis using 0.8% agarose gel. Primers 515F (5′-GTGCCAGCMGCCGCGGTAA-3′) and 907R (5′-CCGTCAATTCMTTTRAGTTT-3′) were used to amplify the 16S rRNA gene [14]. Each 25 μL PCR reaction contains 10 ng DNA, 250 μM dNTPs, 200 nM forward primer, 200 nM reverse primer, 12.5 μg Ambion Ultrapure BSA, FastPfu Buffer, and 1 U of TransStart FastPfu DNA Polymerase (TransGen Biotechnology Co., Ltd., Beijing, China). Cycling conditions were 94 °C for 3 min, followed by 25 cycles of 94 °C for 30 s, 55 °C for 30 s, and 72 °C for 45 s, with a final extension period of 10 min at 72 °C. The resulting PCR products were evaluated by agarose gel electrophoresis. Target bands were recovered using the Qiagen Gel Extraction Kit (Qiagen, Hilden, Germany). Library construction was performed using the TruSeq^®^ DNA PCR-Free Sample Preparation Kit (Illumina, San Diego, CA, USA), and paired-end sequencing was performed using the HiSeq2500 PE250 (Illumina, San Diego, CA, USA).

### 2.6. Statistical Analysis

The raw data obtained by sequencing was controlled by Trimmomatic (version 0.39) software and spliced by FLASH (Adobe Flash CS4 Professional) software, set at a window of 50 bp; the back-end sequences with an average quality of less than 20 bp were removed, and then the sequences with a length of less than 50 bp after quality control (QC) were removed. The sequences at both ends were spliced by overlapping bases, and the sequences with an overlap length of less than 10 bp or a mismatch ratio of greater than 0.2 were filtered with operational taxonomic units (OTUs). The samples were distinguished according to the barcodes and primers at the beginning and end of the sequence, and the sequence orientation was adjusted. The number of mismatches allowed for the barcode was 0. The maximum number of primer mismatches was 2, and the sequence with ambiguous bases was removed to obtain an optimized sequence.

Using UPARSE software (version 7.1 http://drive5.com/uparse/ accessed on 14 November 2023) to perform OTU clustering on the sequences with a similarity of 97% and single sequences, the chimeras were removed during the clustering process to generate an OTU table. Species classification annotation was performed on each sequence using the RDP classifier (https://sourceforge.net/projects/rdp-classifier/ accessed on 14 November 2023) and compared with the Silva database (SSU123) with a comparison threshold of 70%. The α diversity index was calculated with the optimized sequence data: Shannon index, Simpson index, Chao index, etc., and α diversity analysis. FastUniFrac (https://www.majorbio.com/web/ucenter/order/my-order/index accessed on 14 November 2023) was used to analyze the distance matrix between samples to draw a heat map and perform β diversity analysis.

## 3. Result

### 3.1. Effects of Intercropping Medicinal Plants with C. oleifera on Soil Properties and Enzyme Activities

#### 3.1.1. Soil Properties

We aimed to dissect the effects of intercropping medicinal plants on *C. oleifera* soil. The data of the soil physical and chemical properties are shown in Supplementary Table S1. The interplanting patterns EZ, JH, and YDC were found to significantly impact the soil properties. The OM (organic matter) and TN (total nitrogen) were changed maximumly by different intercropping treatments. Compared with the rhizosphere soil of *C. oleifera*, the largest difference occurring in the OM values was observed in JHK, while for TN values, this was YDCY.

The OM of the YDCY treatment in the soil noticeably decreased; however, the TN, pH, NN, TP, AP, and AK considerably increased. The EZ, TN, NN, AP, and OM of the EZY treatment showed significant increases. It can be concluded that the two interplanting patterns EZ and YDC have a noticeable impact on the chemical properties of *C. oleifera* rhizosphere soil, suggesting a potential allelopathic effect on the growth of *C. oleifera* (Table 1).

#### 3.1.2. Soil Enzyme Activities

According to the data of the soil enzyme activities (Table 2), the acid phosphatase (S-ACP) activity of the *C. oleifera* soil was increased in the intercropping with the five medicinal plants, and the highest was observed in the YDC treatment, which was 2.99 mg/g higher than the control treatment (CK). *Clinacanthus nutans*/*C. oleifera* (YDC) and *Ficus simplicissima*/*C. oleifera* (WZMT) significantly increased soil catalase activity (S-CAT). Compared with CKY, the sucrase activity of the JHY and HDKY treatment was extremely increased. *Curcuma longa*/*C. oleifera* (JH) had a critical effect on urease activity (S-UE); the urease content of the three samples was 1.62 mg/g, 1.56 mg/g, and 1.23 mg/g, respectively, which was about twice as much as that of CK. Therefore, it can be concluded that JH and YDC have the most influence on soil enzyme activities.

### 3.2. Effects of Intercropping Medicinal Plants with C. oleifera on Microbial Diversity

Microbial diversity is an important index of the biological community composition. The alpha diversity reflects the abundance and diversity of microorganisms in soil. In this study, the bacteria diversity was measured (Table 3). The results showed that the number of OTU and sequences was sufficient, and the coverage of each sample was more than 99.5%, which indicated that the amount of sequencing data obtained in this study basically reflected the bacterial diversity and composition of each sample. In general, the intercropping patterns had a lower OTU number and less microbial diversity than CK. *Clinacanthus nutans*-roots (YDCY) treatment had the lowest OTU number, which was 714 OTUs less than that of CK. The Shannon index of the YDCY treatment was the lowest (4.63). In contrast, the Simpson index of the soil after intercropping medicinal plants was higher than that of CK. It can be inferred that the intercropping of YDC with *C. oleifera* had a significant impact on the number of OTUs and microbial diversity in the soil. (Table 3).

**Figure 1:**
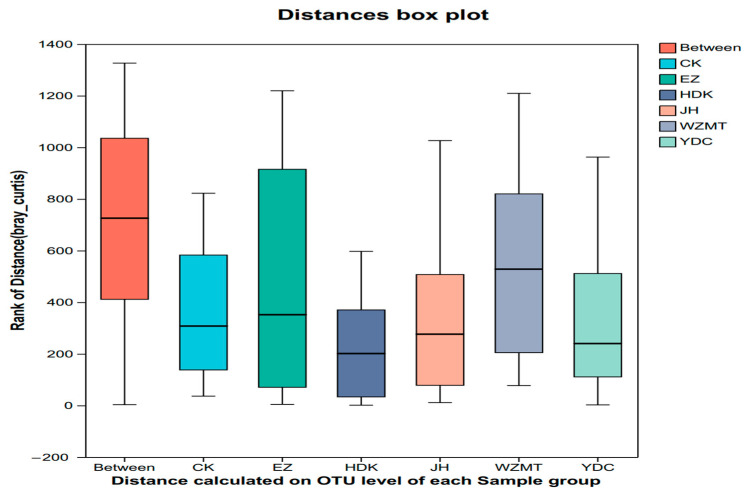
Box diagram of ANOSIM/Adonis analysis at phylum level. CK: Monoculture of *C. oleifera*. EZ: *C. zedoaria*/*C. oleifera*, JH: *C. longa*/*C. oleifera*, YDC: *C. nutans*/*C. oleifera*, HDK: *F. Galangae*/*C. oleifera*, WZMT: *F. simplicissima*/*C. oleifera*.

**Figure 2:**
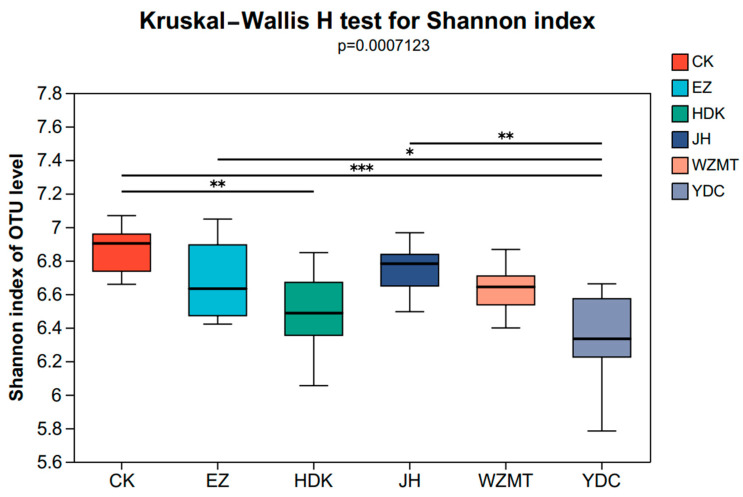
Kruskal–Wallis H test results of Shannon index. The head and tail of a line segment marked with significance indicate the difference between the two samples. *, **, and ***: differences significant at the 0.05, 0.01, and 0.001 levels, respectively.

ANOSIM analysis, known as Analysis of Similarities, is a non-parametric test used to assess whether differences between groups (two or more) are significantly greater than differences within groups, thereby determining if grouping is meaningful. In this study, ANOSIM analysis is used to group three different sampling zones, aiming to investigate whether there are differences in microbial community structure among three different zones. The results indicated no significant differences between groups. Therefore, starting from the large group, that is, the group of different medicinal plants, which included all 96 samples from the three sampling zones, aiming to study the impact of intercropping different medicinal plants on the rhizosphere microbial community structure of *C. oleifera*. The results showed that the median line between the groups was higher than that of the treatment groups and the control group. It was proved that there were differences in microbial diversity among different medicinal plants (Figure 1, Table 4), therefore, the remaining analyses of microbial community structure were conducted using this grouping. Additionally, the study aimed to investigate the effects of one-year and two-year *Curcuma zedoaria* and *Curcuma longa* on *C. oleifera* originally. However, the results indicated no significant differences between them. As a result, the paper only focuses on the five two-year medicinal plants. The Shannon index of the six groups of samples were different at the OTU level (*p* = 0.0007132). There was a significant difference in Shannon index between YDC, HDK and CK, while there was no significant difference between other treatments and CK (Figure 2). 

### 3.3. Effects of Intercropping Medicinal Plants with C. oleifera on Community Structure

#### 3.3.1. Community Structure on OTU Level

**Figure 3:**
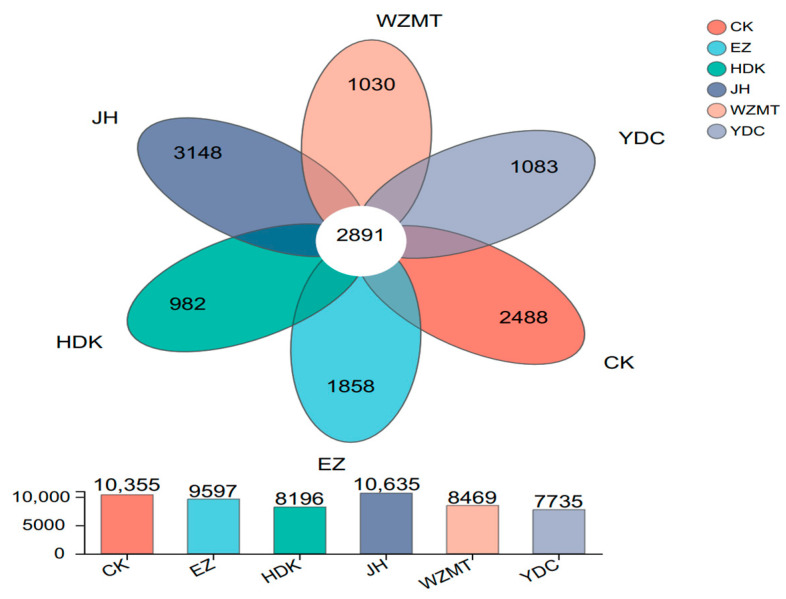
Venn diagram of the composition of bacterial communities in the rhizospheres of *C. oleifera* after intercropping with different medicinal plants. Different colours represent different treatments; the numbers refer to the numbers of species common to multiple treatments in overlapping and non-overlapping sections.

OTU analysis indicated that 2891 OTUs were shared by all samples. The treatment with the largest number of unique OTUs was JH; HDK had the lowest number. The number of OTUs in the JH samples was the highest, 280 more than CK, while YDC had the least, 2620 less than CK. This demonstrated that intercropped JH and YDC had an impact on bacterial diversity. In particular, the OUT number in the YDC treatment declined drastically. This phenomenon may be due to the allelopathic effect of *C. nutans* on *C. oleifera*. (Figure 3).

#### 3.3.2. Community Structure on Genus and Phylum Level

**Figure 4:**
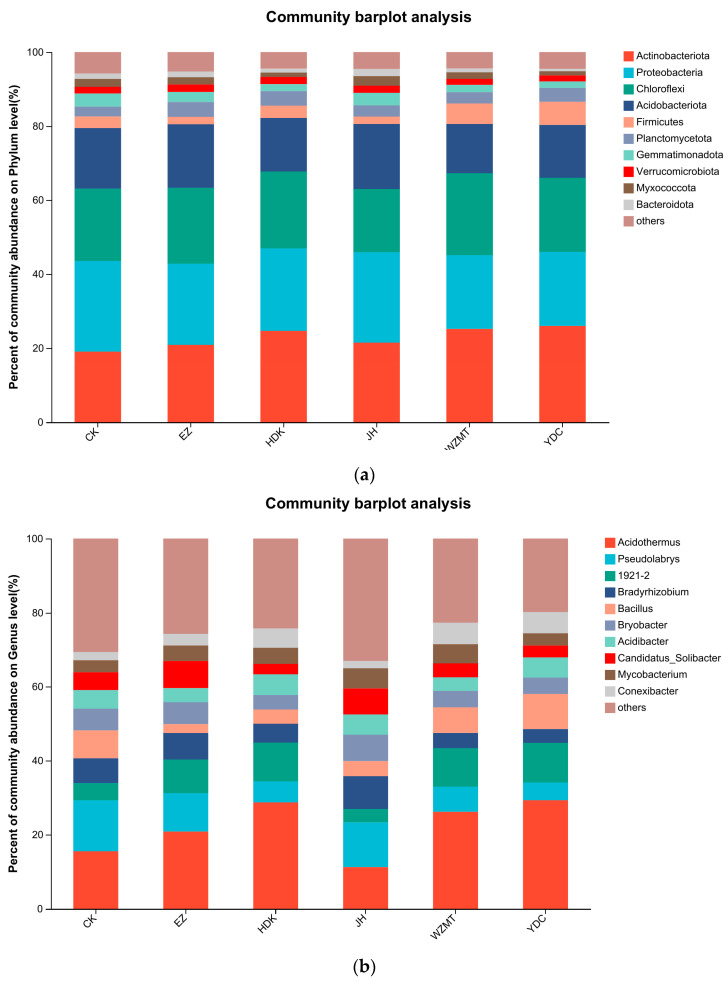
Relative abundance of species composition in different medicinal plants. (**a**) Relative abundance of species composition at phylum level; (**b**) Relative abundance of species composition at genus level. Among the top 50 bacteria of genus level, the top 10 dominant bacteria of existing classification are selected in the figure. CK: Monoculture of *C. oleifera*. EZ: *C. zedoaria*/*C. oleifera*, JH: *C. longa*/*C. oleifera*, YDC: *C. nutans*/*C. oleifera*, HDK: *F. Galangae*/*C. oleifera*, WZMT: *F. simplicissima*/*C. oleifera*.

The community barplot anlysis at the phylum level showed that the structure of dominant bacterial communities in all treatments were the same, whereas the relative abundance of dominant bacteria were different. The top five dominant phyla in the soil were Actinobacteriota, Proteobacteria, Verrucomicrobiota, Acidobacteriota, and Firmicutes. Actinobacteriota and Firmicutes exhibited the highest abundance in the soil intercropped with YDC, while Gemmatimonadota and Myxococcota showed the lowest abundance in YDC. The abundance of Acidobacteriota and Myxococcota in JH was higher than other samples differing from the monoculture of *C. oleifera* in CK (Figure 4a).

At the genus level, community barplot anlysis indicated that the classified dominant bacterial genera included *Acidothermus*, *Pseudoacrobacter*, *Bradyrhizobium*, *Bacillus* and others. Specifically, *Acidothermus*, *1921-2*, *Bacillus*, and *Conexibacter* had higher abundance in YDC than other samples, while *Bradyrhizobium* and *Bryobacter* showed the highest abundance in JH. However, *Bradyrhizobium* had the lowest abundance in YDC. It can be observed that the abundance of bacterial genera had been changed by intercropping with JH and YDC, especially in YDC treatment (Figure 4b).

**Figure 5:**
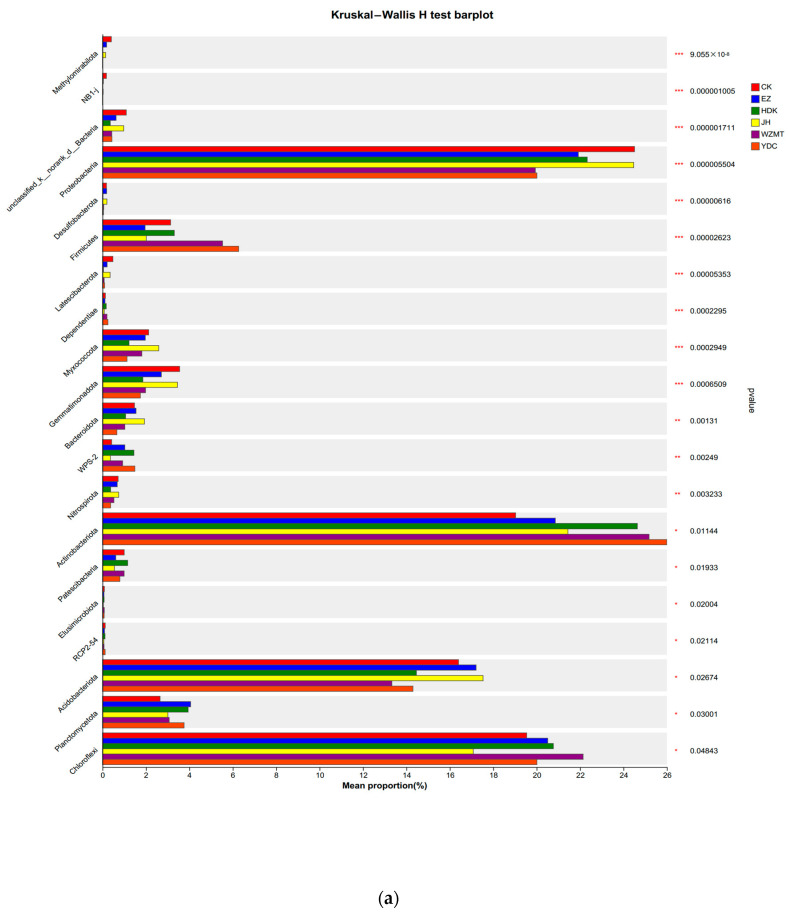

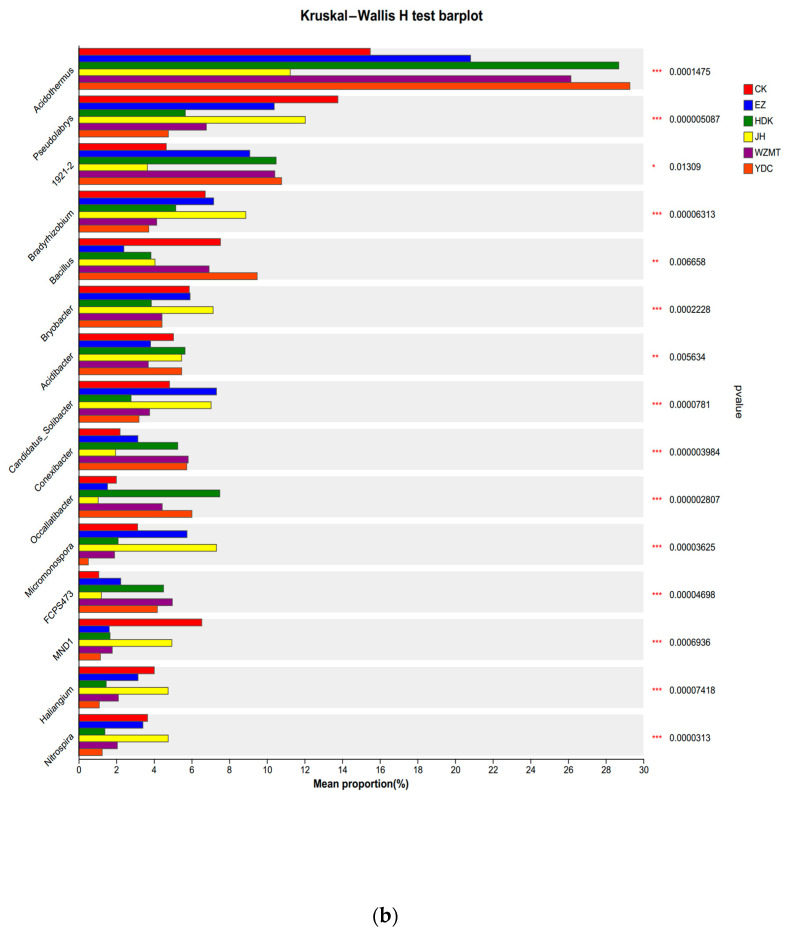
Variations in microbial communities in the rhizospheres of *C. oleifera* after intercropping with different medicinal plants. At a given taxonomic level, the Y-axis represents the species, the X-axis represents the average relative abundance of species in different treatments, and columns of different colours represent different treatments. *, **, and ***: differences significant at the 0.05, 0.01, and 0.001 levels, respectively. (**a**): Between-group significant difference test plot at the phylum level. (**b**): Between-group significant difference test plot at the genus level.

The Kruskal–Wallis H test was conducted to analyze the inter-group differences in the bacterial community composition at the phylum level in different samples. The top twenty bacteria in relative abundance were shown here. There were significant inter-group differences observed in these bacterial communities. Methylomirabilota, NB1-j, and Acidobacteriota in CK were quite different from other samples. Among them, Methylomirabilota, NB1-j, and Acidobacteriota had the highest abundance in CK, while Acidobacteriota had the lowest abundance in CK. Myxococcota, Gemmatimonadota, Bacteroidota, Nitrospirota, Acidobacteriota, and Actinobacteriota displayed significantly higher abundances in JH compared to CK, suggesting an increase in the abundance of these bacteria after intercropping with *C. oleifera*. Particularly, Methylomirabilota, Desulfobacterota, and Latescibacterota were all highly abundant in CK, EZ, and JH, while their abundances in HDK, YDC, and WZMT were extremely low, nearly zero, with highly significant inter-group differences (Figure 5a). 

The inter-group differences in bacterial community composition at the genus level have been displayed in Figure 5b, focusing on the top twenty genera among the fifty species selected at the genus level. Acidothermus showed relatively higher abundance across all groups. Several genera, including *Pseudolabrys*, *Micromonospora*, *MND1*, *Haliangium*, and *Nitrospira*, exhibited the highest abundance in the JH group and the lowest abundance in the YDC group, indicating highly significant inter-group differences. Conversely, *Acidothermus* and *1921-2* showed the opposite trend, with higher abundance in the YDC group and lower abundance in the JH group. These differences were significant compared to CK, revealing a significant shift in soil bacterial community abundance following intercropping with JH and YDC.

**Figure 6:**
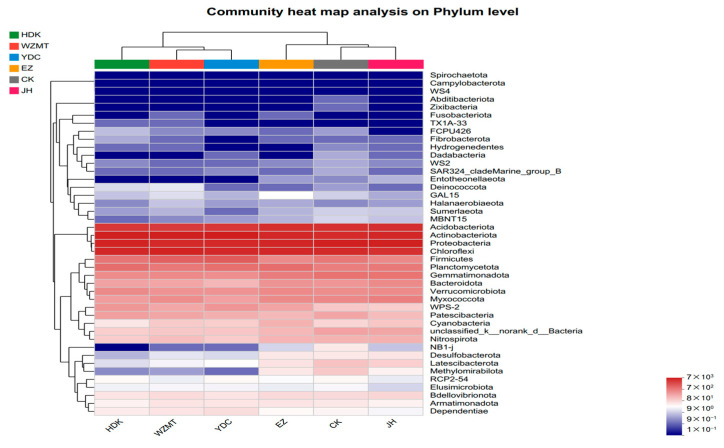
Heat map of correlations between medicinal plants and bacterial communities, with positive correlations in red and negative correlations in blue. EZ: *C. zedoaria*/*C. oleifera*, JH: *C. longa*/*C. oleifera*, YDC: *C. nutans*/*C. oleifera*, HDK: *F. Galangae*/*C. oleifera*, WZMT: *F. simplicissima*/*C. oleifera*, CK: Monoculture of *C. oleifera*.

The heat map of the correlation between samples and bacterial communities at the phylum level reveals that all the experimental groups had a significantly positive correlation with Actinobacteriota, Proteobacteria, Chloroflexi, and Acidobacteriota, and a very significant negative correlation with Spirochaetota and Campylobacterota (Figure 6). NB1-j was positively correlated with CK and negatively correlated with five medicinal plants. Methylomirabilota was significantly negatively correlated with HDK, YDC, and WZMT, but positively correlated with EZ, JH, and CK. The effects of HDK, YDC, and WZMT on bacterial communities were quite different from those of CK. Therefore, these three medicinal plants changed the soil microbial community structure after intercropping with *C. oleifera*. 

### 3.4. Correlation Analysis of Soil Properties, Enzyme Activities and Soil Microbiome

**Figure 7:**
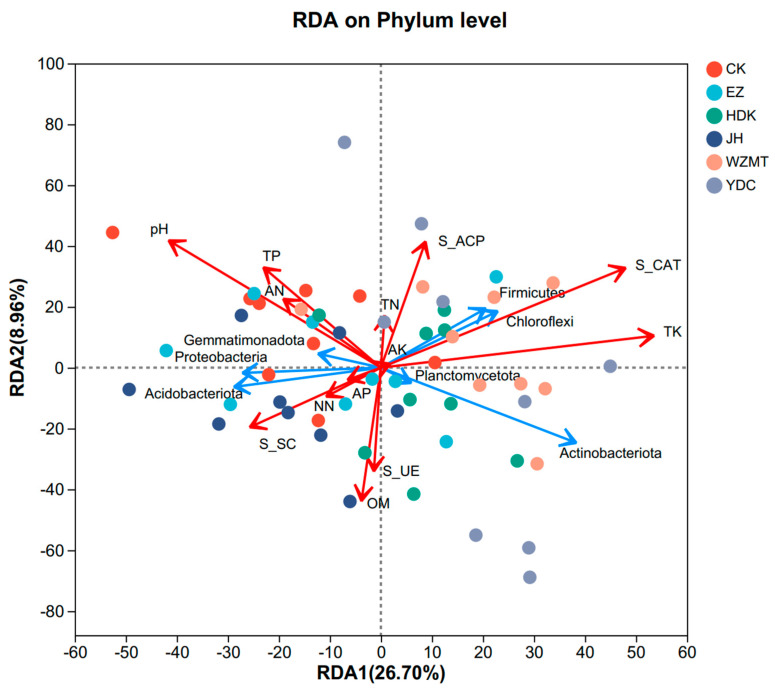
Redundancy analyses (RDA) of bacterial communities and soil properties and enzyme activities in five medicinal plants—*C. oleifera* intercropping systems. OM—organic matter, TN—total nitrogen, TP—total phosphorus, TK—total potassium, AN—ammonium nitrogen, NN—nitrate nitrogen, AP—available phosphorus, AK—available potassium, S-UE—soil urease activity, S-CAT—soil catalase activity, S-SC—soil sucrase activity, S-ACP—soil acid phosphatase activity. EZ: *C. zedoaria*/*C. oleifera*, JH: *C. longa*/*C. oleifera*, YDC: *C. nutans*/*C. oleifera*, HDK: *F. Galangae*/*C. oleifera*, WZMT: *F. simplicissima*/*C. oleifera*, CK: Monoculture of *C. oleifera*.

Redundancy analysis (RDA) was used to further analyze the effects of soil chemical properties and enzyme activities on the soil microbial community structure. The explanatory power of the first axis RDA1 was 26.70%, the second axis was 8.96%, and the total explanatory degree was 35.66%. The first and second axes accounted for 26.70% and 8.96%, respectively, of the total variation between soil properties and bacterial community composition. Soil catalase activity (r^2^ = 0.3995, *p* = 0.001), total potassium (r^2^ = 0.3437, *p* = 0.001), and pH (r^2^ = 0.2324, *p* = 0.001) were the most important factors affecting the soil bacterial community. Soil catalase activity and total potassium were positively correlated with Chloroflexi, Acidobacteriota, and Firmicutes, and negatively correlated with Gemmatimonadota, Acidobacteriota, and Proteobacteria. In addition, soil pH was positively correlated with Gemmatimonadota, Acidobacteriota, and Proteobacteria, and negatively correlated with Actinobacteriota, Firmicutes, and Chloroflexi (Figure 7).

## 4. Discussion

### 4.1. Effects on Soil Chemical Properties and Enzyme Activities after Intercropping Medicinal Plants with C. oleifera

Soil nutrients play a crucial role in the growth of plants, and soil enzymes are biological catalysts produced in the soil that participate in the transformation of organic compounds and the decomposition of plant and animal residues, serving as important indicators of soil fertility [15,16]. Our research results indicate that compared to the control (CK), the intercropping systems EZ, JH, and YDC all increased the content of total nitrogen, available phosphorus, and available potassium in the rhizosphere soil of *C. oleifera* while decreasing the pH. This result is consistent with that of a study reporting that in a wheat/faba bean intercropping system, the rhizosphere pH decreased, but the rhizosphere P availability increased compared with monocropped faba beans and wheat [6]. However, the results are different from those of Sujatha and Song [17,18].

In our research, the contents of total nitrogen, nitrate nitrogen, and available potassium in *C. oleifera* rhizosphere soil were increased in the treatments intercropped with medicinal plants. Among them, YDC had the greatest impact on the total nitrogen content in *C. oleifera* rhizosphere soil, increasing the content by 7.82 g/kg, and JH had the greatest impact on nitrate nitrogen, increasing it by 722.33 mg/kg. The decrease in ammonium nitrogen content in the YDC treatment group may be due to the nitrification process, converting ammonium nitrogen into nitrate nitrogen, which is less prone to loss and volatilization, thereby increasing the total nitrogen content [19,20]. The available potassium content in the *C. oleifera* rhizosphere soil increased significantly in the JH treatment. This may be because *C. longa* itself is a medicinal plant rich in minerals such as potassium and calcium [21]; that is to say, the cultivar characteristics of intercropping herbs may lead to changes in the chemical properties of the rhizosphere soil [22].

Based on the changes of the physicochemical properties of the *C. oleifera* rhizosphere soil intercropped with different medicinal plants, it can be concluded that EZ, JH, and YDC have obvious effects on rhizosphere soil physicochemical properties and may have allelopathic effects on the growth of *C. oleifera*. The EZ, JH, and YDC treatments were significantly different from the other three treatments, i.e., CK, WZMT, and HDK, in a variety of chemical properties and soil enzyme activities. Interestingly, the organic matter content of the YDC intercropping pattern significantly decreased compared to CK, while the total nitrogen increased. This contradicts a general view that a decrease in organic matter leads to a decrease in total nitrogen, suggesting that the increased nitrogen may be inorganic nitrogen [23,24]. In summary, intercropping with EZ, JH, and YDC has a significant impact on the chemical properties of the *C. oleifera* rhizosphere soil, indicating a potential allelopathic effect on the growth of *C. oleifera*.

Catalase is an important redox enzyme in the soil, and its function is to break down the toxic hydrogen peroxide free radicals for organisms [25,26]. The activity of soil catalase (S-CAT) was increased significantly in the YDC and WZMT treatments, each increased by 2.27 mg/g and 3.97 mg/g. Researchers have shown that metal ions, proteins, and vitamins can promote catalase activity. *Clinacanthus nutans*/*C. oleifera* (YDC) and *Ficus simplicissima*/*C. oleifera* (WZMT) are rich in proteins, vitamins, and polyphenols [27,28,29], which may contribute to the increased catalase activity of the *C. oleifera* rhizosphere soil. In our research, five medicinal plants increased the activity of soil acid phosphatase (S-ACP) in the *C. oleifera* rhizosphere soil. Chen and Margalef [30,31] confirmed the close correlation between soil phosphatase and soil-available phosphorus. Phosphatase plays a crucial role in the process of converting inorganic phosphorus into organic phosphorus, and organic phosphorus can be absorbed by plants [32]. The increase in soil phosphatase activity can promote the activation of phosphorus [33,34]. Thus, the soil-available phosphorus content is higher. The research results are consistent with those of studies on the intercropping of wolfberry and gramineae plants [35], intercropping with walnut and tea [36], and intercropping with peanut under tea [37]. Xiao [38] found that intercropping cucumber with green garlic increased soil catalase and alkaline phosphatase activities, which aligns with our research results. Sucrase activity can influence the transformation of organic matter. In our research, sucrase activity was increased in EZ, JH, and HDK, indicating that intercropping with these three medicinal plants can promote *C. oleifera* rhizosphere soil fertility. Urease is a hydrolytic enzyme that promotes the hydrolysis of chemical bonds in organic compounds in the soil, leading to the generation of ammonia. This ammonia can serve as one of the sources of nitrogen for plants [39]. There was a significant increase in urease (S-UE) activity and total nitrogen (TN) content in JH treatment. This may be because the root exudates of JH can promote nitrogen conversion. In conclusion, JH and YDC have the most significant impact on soil enzyme activities.

### 4.2. Effects on Soil Microbial Diversity and Structure after Intercropping Medicinal Plants with C. oleifera

#### 4.2.1. Soil Microbial Diversity

The rhizosphere soil microorganisms, as an important component of the soil ecosystem, play critical roles in soil material cycling, energy metabolism, plant growth and development, disease resistance, and stress tolerance [40]. Compared to monocropping, the number of OTUs and Shannon index showed a decreasing trend in the medicinal plant—*C. oleifera* intercropping systems. This suggests a reduction in species richness and microbial diversity after intercropping, with the most significant impact attributed to YDC, followed by WZMT. In other studies, it has also been found that microbial diversity decreases after intercropping, such as intercropping alfalfa with mulberry trees [9] and green manure plants in lychee orchards [41]. In the Shannon index intergroup difference test, there were significant differences in the Shannon index between YDC, HDK, and CK. This was mainly because microbial growth was inhibited by root exudate changes due to the increased nitrogen content [42].

#### 4.2.2. Soil Microbial Community Structure

Venn can be used to statistically analyze the number of common and unique species or OTUs among samples, providing a visually intuitive representation of the similarity and overlap of species or OTUs. Sample JH had the highest number of unique OTUs, whilst YDC had the lowest. The results indicated that there were differences in the effects of intercropping different medicinal plants with *C. oleifera* on soil microorganisms, and JH may have played a role in enriching microorganisms, while YDC may have a certain inhibitory effect.

According to the comparative analysis using PCoA and intergroup community difference tests of soil microbial communities after intercropping, the CK, EZ, and JH groups were distinctly separated from the HDK, YDC, and WZMT groups. Methylomirabilota, Desulfobacterota, and Latescibacterota may be the main factors causing the differences.

The relative abundance of Actinobacteriota and Firmicutes was increased in the YDC treatment. Some kinds of bacteria classified into Actinobacteria and Firmicutes can promote plant growth, inhibiting the growth of pathogens [43,44,45]. Comparing JH treatment with monocropping, the relative abundance of Acidobacteriota and Myxococcota was increased. Acidobacteria play an essential role in carbon cycling via vitamins and other polysaccharides decomposing [46]. They are also involved in iron cycling and promote photosynthesis [47]. Methylomirabilota was not identified in the YDC intercropping treatment soil. Soil nitrogen is an important factor influencing the composition and structure of methane-oxidizing bacterial communities [48]. The nitrogen content and its different forms can affect methane-oxidizing bacteria [49]. In the correlation heat map between different samples and bacterial communities, NB1-J was the only bacterium that was positively associated with CK, but negatively associated with all five medicinal plants. HDK, WZMT, and YDC showed a significant negative correlation with Methylomirabilota, while they were positively correlated with CK, EZ, and JH. The correlations of EZ and JH with other bacteria were also consistent with CK. Therefore, the soil microbial community structure was affected by intercropping treatments, i.e., HDK, YDC, and WZMT.

### 4.3. The Correlation between Soil Chemical Properties, Enzyme Activities, and Soil Microbial Community

The RDA results indicated that the microbial community was mainly affected by S-CAT, TK, and pH. In Chen’s [50] research, catalase activity was discussed as a major environmental factor affecting the Proteobacteria phylum. In our research, a negative correlation was observed between catalase activity and the Proteobacteria, consistent with previous research. The hydrogen peroxide enzyme activity of the soil showed a highly significant negative correlation with the Acidobacteria, same with Jiang [51] and Gao [52] and other results. The pH values were positively correlated with several bacterial phyla [53,54,55]. Soil pH can induce changes in the composition and abundance of soil microbial communities by affecting the chemical fertility characteristics of the soil matrix [56]. The potassium content in the soil is crucial for plant resistance [57], determining crop productivity and quality. Vuong’s [58] study indicated a significant correlation between total potassium and available potassium within fungal and bacterial communities. Wan’s [59] research mentioned a significant correlation between TK and Actinobacteria, consistent with our study. Most dominant bacteria showed a negative correlation with nitrogen, phosphorus, and potassium, indicating that soil factors influence the structure of microbial communities. The JH and YDC treatments were concentrated on the dominant bacterial groups in the RDA analysis, suggesting that intercropping the two medicinal plants with *C. oleifera* may have a certain impact on the microbial community of the rhizosphere soil.

## 5. Conclusions

This study mainly investigated the changes of the soil chemical properties, enzyme activities, and microbial community structure of *C. oleifera*–medicinal plant intercropping systems. The results indicated that intercropping five medicinal plants with *C. oleifera* significantly increased the content of soil factors such as TN, NN, and AK, as well as enzyme activities, particularly in the JH and YDC treatments. However, intercropping with medicinal plants led to a decrease in soil microbial diversity, with the YDC intercropping group showing the lowest microbial diversity. Medicinal plants enhanced the relative abundance of dominant bacterial groups. *Clinacanthus nutans*/*C. oleifera* (YDC), *Fructus Galangae*/*C. oleifera* (HDK), and *Ficus simplicissima*/*C. oleifera* (WZMT) significantly altered the microbial community structure. Soil pH, TK, and S-CAT were identified as the main factors influencing the microbial community structure. In conclusion, the soil nutrients and microbial community structure of the JH and YDC intercropping treatments changed greatly, and intercropping these two medicinal plants with *C. oleifera* could be considered in cultivation. This study provides theoretical guidance for *C. oleifera* agroforestry systems.

## Figures and Tables

**Table 1:** Soil chemical properties.

Samples	pH	OM (g/kg)	TN (g/kg)	NN (mg/kg)	AN (mg/kg)	TP (g/Kg)	AP (mg/kg)	TK (g/kg)	AK (mg/kg)
CKZ	5.35 ± 0.59	15.43 ± 1.69	1.84 ± 0.42	161.67 ± 8.08	427.67 ± 10.07	2.7 ± 0.54 *	50.53 ± 1.69	3.58 ± 0.27	18.13 ± 8.74 **
CKK	5.37 ± 0.26	15 ± 1.14	1.87 ± 0.2	124 ± 11.53	426.67 ± 12.66	4.47 ± 0.41	49.73 ± 4.22	3.09 ± 0.21	7.97 ± 0.4
CKY	5.89 ± 0.07	13.77 ± 1.76 **	1.27 ± 0.04 **	109.67 ± 7.64 **	112.33 ± 4.04 **	4.02 ± 1.24	48.07 ± 3.95	2.91 ± 0.44 *	9.23 ± 3.8
EZZ	4.43 ± 0.27	15.1 ± 0.36	4.17 ± 0.35	537 ± 21.63 *	362.67 ± 4.73	3.17 ± 0.95	53.27 ± 6	3.71 ± 0.19	19.8 ± 7.63
EZK	4.26 ± 0.24	15.3 ± 0.26	1.28 ± 0.11 **	593.67 ± 20.21 *	596.33 ± 6.66 **	3.29 ± 0.14	47.9 ± 9.56	3.45 ± 0.22	11.3 ± 1.78
EZY	5.46 ± 0.54 **	16.13 ± 0.35 *	6.66 ± 1.08 **	832 ± 51.97 **	212 ± 9.54	4.21 ± 0.97	76.5 ± 16.85 **	3.45 ± 0.1	21.67 ± 1.29 **
JHZ	4.29 ± 0.15	15.93 ± 0.38	3.75 ± 0.48	258.33 ± 3.21	132 ± 6.08	3.33 ± 0.08	79.07 ± 14.17	2.82 ± 0.34	20.3 ± 2.91
JHK	3.91 ± 0.07 **	16.47 ± 0.06 **	1.9 ± 0.2	250.67 ± 18.23	99.67 ± 4.04	2.91 ± 0.33	57.07 ± 1.72 *	2.8 ± 0.13	19.97 ± 4.45
JHY	4.71 ± 0.05	15.47 ± 0.47	7.13 ± 0.31 **	245 ± 12.77	107 ± 2.65	4.02 ± 0.94 *	73.93 ± 4.58	2.85 ± 0.1	32.5 ± 5.15 **
HDKZ	4.02 ± 0.05	16.33 ± 0.21 **	2.11 ± 0.17	342.67 ± 22.23	96.33 ± 2.52	1.76 ± 0.25	45.87 ± 2.33	3.44 ± 0.08	18.13 ± 0.7
HDKK	3.99 ± 0.08	15.63 ± 0.57	3.55 ± 0.14	373.67 ± 10.21	132.67 ± 8.39	2.46 ± 0.18	49.63 ± 1.7	3.62 ± 0.21	17.67 ± 2.87
HDKY	4.82 ± 0.14 *	15.3 ± 0.17	2.33 ± 0.19	291.33 ± 65.58	190.67 ± 1.53 *	2.71 ± 0.12	44.37 ± 4.25	3.73 ± 0.11	30.1 ± 4.09 *
YDCZ	4.14 ± 0.07	15.53 ± 0.61	1.85 ± 0.24	254 ± 28.35	93 ± 3.61	2.46 ± 0.21	70.9 ± 1.42	3.87 ± 0.03	20.9 ± 1.11
YDCK	4.1 ± 0.12	15.97 ± 0.8	2.37 ± 0.24	232 ± 1.73	99.33 ± 2.52	2.55 ± 0.45	58.47 ± 9.6	4.59 ± 0.33 *	20.8 ± 5.31
YDCY	4.75 ± 0.25 *	12.87 ± 0.75 **	9.09 ± 0.33 **	345 ± 50.48 *	94.67 ± 6.03	4.39 ± 0.7 *	89.1 ± 11.17 *	4.16 ± 0.64	31.17 ± 12.98 **
WZMTZ	4.37 ± 0.08	16.4 ± 0.4 **	3.16 ± 0.21	224 ± 9.54	118.33 ± 4.51	2.21 ± 0.15	52.6 ± 0.95	4.15 ± 0.43	18.4 ± 1.3
WZMTK	4.53 ± 0.42	15.23 ± 0.38	3.25 ± 0.36	240.67 ± 5.69	73 ± 3.61 **	2.37 ± 0.25	45.83 ± 0.51	4.28 ± 0.1	14.53 ± 0.87
WZMTY	4.74 ± 0.18	15.77 ± 0.61	5.36 ± 0.25 *	199 ± 24.25	203.33 ± 5.51 *	3.6 ± 1.27 *	61.17 ± 4.5 *	3.99 ± 0.45	21.17 ± 1.16 *

The data were evaluated by one-way ANOVA, followed by the Tukey-HSD test at 0.05 to determine significant differences indicated by * (mean ± SD, *n* = 96). **: significantly correlated at the 0.01 level, *: significantly correlated at the 0.05 level. Abbreviations: OM—organic matter, TN—total nitrogen, TP—total phosphorus, TK—total potassium, AN—ammonium nitrogen, NN—nitrate nitrogen, AP—available phosphorus, AK—available potassium. EZZ: C.zed.-cover roots, EZK: C.zed.-C.ole. soil, EZY: C.ole.-cover roots. JHZ: C. lon.-cover roots, JHK: C. lon.-C. ole. soil, JHY: C.ole.-cover roots. YDCZ: C. nut.-C. ole. soil ,YDCK: C. nut.-cover roots, YDCY: C.ole.-cover roots. HDKZ: F. Gal./C. ole.-soil, HDKK: F. Gal.-cover roots, HDKY: C.ole.-cover roots. WZMTZ: F. sim.-C. ole. soil, WZMTK: F. sim.-cover roots, WZMTY: C.ole.-cover roots. CKZ: C.ole.-cover roots, CKK: C.ole.- C.ole. soil, CKY: C.ole.-cover roots.

**Table 2:** Soil enzyme activities.

Samples	S-CAT (mg/g)	S-ACP (mg/g)	S-SC (mg/g)	S-UE (mg/g)
CKZ	5.05 ± 0.59	1.01 ± 0.03	2.3 ± 0.27	0.73 ± 0.08
CKK	5.79 ± 0.75	1.59 ± 0.05	1.86 ± 0.06	0.47 ± 0.08
CKY	5.42 ± 0.17	2.46 ± 0.37 *	1.43 ± 0.04	0.56 ± 0.03
EZZ	4.46 ± 0.52	1.58 ± 0.09	7.24 ± 0.11	1.02 ± 0.05 *
EZK	5.26 ± 0.32 *	2.57 ± 0.11	6.48 ± 0.27	0.57 ± 0.06
EZY	4.12 ± 0.84	3.54 ± 0.15 *	6.79 ± 0.28	0.66 ± 0.05
JHZ	4.07 ± 0.48	1.54 ± 0.12	3.87 ± 0.43	1.62 ± 0.06 **
JHK	2.49 ± 0.29 **	2.64 ± 0.16	4.88 ± 0.37	1.56 ± 0.1 **
JHY	4.22 ± 0.26	3.6 ± 0.04 *	9.15 ± 0.75 **	1.23 ± 0.09 *
HDKZ	4.45 ± 0.37	1.55 ± 0.1	2.96 ± 0.14	1.26 ± 0.07
HDKK	4.28 ± 0.09	2.4 ± 0.08	3.01 ± 0.11	0.77 ± 0.08
HDKY	5.59 ± 1.05 *	3.78 ± 0.22 *	6.29 ± 0.77 **	1.53 ± 0.05
YDCZ	5.87 ± 0.72	2.14 ± 0.05	3.08 ± 0.1	1.1 ± 0.07
YDCK	4.41 ± 0.4	3.39 ± 0.03	3.02 ± 0.37	1.02 ± 0.01
YDCY	7.69 ± 0.46 **	5.45 ± 0.19 **	2.72 ± 0.27	0.82 ± 0.09
WZMTZ	8.02 ± 0.63	1.56 ± 0.13	3.44 ± 0.2	0.85 ± 0.05
WZMTK	8.46 ± 0.64	2.42 ± 0.14	3.74 ± 0.68	0.89 ± 0.06
WZMTY	9.39 ± 0.88 *	3.56 ± 0.12 *	2.94 ± 0.76	0.68 ± 0.1

The data were evaluated by one-way ANOVA, followed by the Tukey-HSD test at 0.05 to determine significant differences indicated by * (mean ± SD, *n* = 96). **: significantly correlated at the 0.01 level, *: significantly correlated at the 0.05 level. Abbreviations: S-UE—soil urease activity, S-CAT—soil catalase activity, S-SC—soil sucrase activity, S-ACP—soil acid phosphatase activity. EZZ: C.zed.-cover roots, EZK: C.zed.-C.ole. soil, EZY: C.ole.-cover roots. JHZ: C. lon.-cover roots, JHK: C. lon.-C. ole. soil, JHY: C.ole.-cover roots. YDCZ: C. nut.-C. ole. soil, YDCK: C. nut.-cover roots, YDCY: C.ole.-cover roots. HDKZ: F. Gal./C. ole.-soil, HDKK: F. Gal.-cover roots, HDKY: C.ole.-cover roots. WZMTZ: F. sim.-C. ole. soil, WZMTK: F. sim.-cover roots, WZMTY: C.ole.-cover roots. CKZ: C.ole.-cover roots, CKK: C.ole.- C.ole. soil, CKY: C.ole.-cover roots.

**Table 3:** Alpha diversity index of rhizosphere soil microorganisms under five medicinal plants—*C. oleifera* intercropping conditions.

Sample	OTU_Num	Sequences	Sobs	Shannon	Simpson	Ace	Chao	Coverage
CKZ	3617	25,851	644	4.95	0.015	751	750	0.995
CKK	3106	25,851	600	4.9	0.014	705	718	0.995
CKY	3256	25,851	625	4.99	0.013	712	714	0.996
EZZ	3447	25,851	634	5.01	0.013	746	758	0.995
EZK	2748	25,851	498	4.44	0.025	569	567	0.996
EZY	2989	25,851	588	4.87	0.017	663	668	0.996
JHZ	3368	25,851	632	5	0.014	723	723	0.995
JHK	3005	25,851	565	4.87	0.016	649	650	0.996
JHY	3239	25,851	587	4.9	0.016	661	674	0.996
HDKZ	2679	25,851	539	4.6	0.022	631	633	0.996
HDKK	2269	25,851	470	4.32	0.03	554	553	0.996
HDKY	3132	25,851	585	4.86	0.017	676	691	0.996
YDCZ	2193	25,851	454	4.26	0.035	537	543	0.996
YDCK	2600	25,851	520	4.52	0.024	617	617	0.996
YDCY	2542	25,851	525	4.63	0.022	609	604	0.996
WZMTZ	2970	25,851	614	4.9	0.016	713	729	0.995
WZMTK	2254	25,851	532	4.45	0.029	609	606	0.996
WZMTY	2678	25,851	541	4.73	0.019	624	640	0.996

Abbreviations: EZZ: C.zed.-cover roots, EZK: C.zed.-C.ole. soil, EZY: C.ole.-cover roots. JHZ: C. lon.-cover roots, JHK: C. lon.-C. ole. soil, JHY: C.ole.-cover roots. YDCZ: C. nut.-C. ole. soil ,YDCK: C. nut.-cover roots, YDCY: C.ole.-cover roots. HDKZ: F. Gal./C. ole.-soil, HDKK: F. Gal.-cover roots, HDKY: C.ole.-cover roots .WZMTZ: F. sim.-C. ole. soil, WZMTK: F. sim.-cover roots, WZMTY: C.ole.-cover roots. CKZ: C.ole.-cover roots, CKK: C.ole.-C.ole. soil, CKY: C.ole.-cover roots.

**Table 4:** ANOSIM analysis results.

Method	Statistic	*p* Value	Permutation Number
ANOSIM	0.503	0.001	999

## Data Availability

The data presented in this study are available on request from the corresponding author. The data are not publicly available due to the policy of the institute.

## Supplementary Materials

The following supporting information can be downloaded at: https://www.mdpi.com/article/10.3390/microorganisms12081616/s1, Table S1. Soil physical properties.

## References

[1] Liu J., Wu L.C., Chen D., Yu Z.G., Wei C.J. (2018). Development of a soil quality index for *Camellia oleifera* forestland yield under three different parent materials in Southern China. Soil Tillage Res..

[2] Lu W.T., Shen X.F., Chen Y. (2019). Effects of intercropping peanut on soil nutrient status and microbial activity within young *Camellia oleifera* plantation. Commun. Soil Sci. Plant Anal..

[3] Tu J., Chen J.F., Zhou J.H., Ai W.S., Chen L.S. (2019). Plantation quality assessment of *Camellia oleifera* in mid-subtropical China. Soil Tillage Res..

[4] Li J., Wu Z.L., Yuan J. (2019). Impact of Agro-Farming Activities on Microbial Diversity of Acidic Red Soils in a *Camellia Oleifera* Forest. Rev. Bras. Ciencia Solo.

[5] Rizvi S.J.H., Tahir M., Rizvi V., Kohli R., Ansari A. (1999). Allelopathic interactions in agroforestry systems. Crit. Rev. Plant Sci..

[6] Zhang X.P., Gao G.B., Wu Z.Z., Wen X., Zhong H., Zhong Z.Z., Yang C.B., Bian F.Y., Gai X. (2020). Responses of soil nutrients and microbial communities to intercropping medicinal plants in moso bamboo plantations in subtropical China. Environ. Sci. Pollut. Res..

[7] Liu C.Z., Cai Q.Z., Liao P.R., Jiang X.L., Tang X.M., Yang Q., Zhou L.Y. (2021). Effects of *Fallopia multiflora*–*Andrographis paniculata* intercropping model on yield, quality, soil nutrition and rhizosphere microorganisms of *F. multiflora*. Plant Soil.

[8] Crowder D.W., Northfield T.D., Strand M.R., Snyder W.E. (2010). Organic agriculture promotes evenness and natural pest control. Nature.

[9] Li M.Z., Wei Y.W., Yin Y., Zhu W.X., Bai X.J., Zhou Y.B. (2023). Characteristics of Soil Physicochemical Properties and Microbial Community of Mulberry (*Morus alba* L.) and Alfalfa (*Medicago sativa* L.) Intercropping System in Northwest Liaoning. Microorganisms.

[10] Zhang Y., Lei J.J., Peng Y.Y., Chen X.Y., Li B.W., Chen Y.Z., Xu Y.C., Farooq T.H., Wu X.H., Wang J. (2024). Impact of Intercropping on Nitrogen and Phosphorus Nutrient Loss in *Camellia oleifera* Forests on Entisol Soil. Forests.

[11] Duan Y.H.Z., Zhou T., He Z., Peng Y.Y., Lei J.J., Dong J.Y., Wu X.H., Wang J., Yan W.D. (2023). Effects of Straw Mulching on Soil Properties and Enzyme Activities of *Camellia oleifera*–Cassia Intercropping Agroforestry Systems. Plants.

[12] Bao S.D. (2000). Analysis Method of Soil and Agricultural Chemistry.

[13] Guan S.Y. (1986). Soil Enzymology and Its Methodology.

[14] Hassan A., Din A.U., Zhu Y., Zhang K., Li T., Wang Y., Xu S., Lei H., Yu X., Wang G. (2020). Anti-atherosclerotic effects of *Lactobacillus plantarum* ATCC 14917 in ApoE^−/−^ mice through modulation of proinflammatory cytokines and oxidative stress. Appl. Microbiol. Biotechnol..

[15] Utobo E.B., Tewari L. (2015). Soil Enzymes as Bioindicators of Soil Ecosystem Status. Appl. Ecol. Environ. Res..

[16] Huang X.M., Muneer M.A., Li J., Hou W., Ma C.C., Jiao J.B., Cai Y.Y., Chen X.H., Wu L.Q., Zheng C.Y. (2021). Integrated Nutrient Management Significantly Improves Pomelo (*Citrus grandis*) Root Growth and Nutrients Uptake under Acidic Soil of Southern China. Agronomy.

[17] Sujatha S., Bhat R., Kannan C., Balasimha D. (2011). Impact of intercropping of medicinal and aromatic plants with organic farming approach on resource use efficiency in arecanut (*Areca catechu* L.) plantation in India. Ind. Crops Prod..

[18] Song Y.N., Zhang F.S., Marschner P., Fan F.L., Gao H.M., Bao X.G., Sun J.H., Li L. (2007). Effect of intercropping on crop yield and chemical and microbiological properties in rhizosphere of wheat (*Triticum aestivum* L.), maize (*Zea mays* L.), and faba bean (*Vicia faba* L.). Biol. Fertil. Soils.

[19] Antil R., Lovell R., Hatch D., Jarvis S. (2001). Mineralization of nitrogen in permanent pastures amended with fertilizer or dung. Biol. Fertil. Soils.

[20] Hartmann A.A., Barnard R.L., Marhan S., Niklaus P.A. (2013). Effects of drought and N-fertilization on N cycling in two grassland soils. Oecologia.

[21] Yang X., Yang Z.L., Liu R.N., Wang J. (2011). Location variation of curcuminoids content in *Curcuma longa* rhizome and CCA analysis with environmental factors. J. Plant Resour. Environ..

[22] Jabborova D., Choudhary R., Karunakaran R., Ercisli S., Ahlawat J., Sulaymanov K., Azimov A., Jabbarov Z. (2021). The Chemical Element Composition of Turmeric Grown in Soil-Climate Conditions of Tashkent Region, Uzbekistan. Plants.

[23] Ros G.H. (2012). Predicting soil N mineralization using organic matter fractions and soil properties: A re-analysis of literature data. Soil Biol. Biochem..

[24] Miles N., Van Antwerpen R., Ramburan S. (2017). Soil organic matter under sugarcane: Levels, composition and dynamics. Int. Sugar J..

[25] Bandick A.K., Dick R.P. (1999). Field management effects on soil enzyme activities. Soil Biol. Biochem..

[26] Zimmermann S., Frey B. (2002). Soil respiration and microbial properties in an acid forest soil: Effects of wood ash. Soil Biol. Biochem..

[27] Devasvaran K., Alallam B., Lim V. (2023). Optimisation of the extraction of crude polysaccharides from *Clinacanthus nutans* leaves for antioxidant applications: Content analysis, chemometrics and metabolomics analysis. Ind. Crops Prod..

[28] Alam M.A., Zaidul I.S.M., Ghafoor K., Sahena F., Hakim M.A., Rafii M.Y., Abir H.M., Bostanudin M.F., Perumal V., Khatib A. (2017). In vitro antioxidant and, α-glucosidase inhibitory activities and comprehensive metabolite profiling of methanol extract and its fractions from *Clinacanthus nutans*. BMC Complement. Altern. Med..

[29] Uddin M.K., Shamsuzzaman S.M., Lo L.Q., Medom M., Hasan M. (2017). Effects of salinity on growth, antioxidant contents and proximate compositions of sabah snake grass (*Clinacanthus nutans* (burm. F.) Landau). Bangladesh J. Bot..

[30] Chen X.L., Chen H.Y.H., Chang S.X. (2022). Meta-analysis shows that plant mixtures increase soil phosphorus availability and plant productivity in diverse ecosystems. Nat. Ecol. Evol..

[31] Margalef O., Sardans J., Fernández-Martínez M., Molowny-Horas R., Janssens I.A., Ciais P., Goll D., Richter A., Obersteiner M., Asensio D. (2017). Global patterns of phosphatase activity in natural soils. Sci. Rep..

[32] Shi S., Tian L., Ma L., Tian C. (2018). Community Structure of Rhizomicrobiomes in Four Medicinal Herbs and Its Implication on Growth Management. Microbiology.

[33] Hui D.F., Mayes M.A., Wang G.S. (2013). Kinetic parameters of phosphatase: A quantitative synthesis. Soil Biol. Biochem..

[34] Qu X.J., Liao Y.W.K., Pan C., Li X.G. (2024). Positive effects of intercropping on soil phosphatase activity depend on the application scenario: A meta-analysis. Soil Tillage Res..

[35] Zhu L.Z., He J., Tian Y., Li X.Y., Li Y.H., Wang F., Qin K., Wang J. (2022). Intercropping Wolfberry with Gramineae plants improves productivity and soil quality. Sci. Hortic..

[36] Bai Y.C., Li B.X., Xu C.Y., Raza M., Wang Q., Wang Q.Z., Fu Y.N., Hu J.Y., Imoulan A., Hussain M. (2022). Intercropping Walnut and Tea: Effects on Soil Nutrients, Enzyme Activity, and Microbial Communities. Front. Microbiol..

[37] Farooq T.H., Kumar U., Mo J., Shakoor A., Wang J., Rashid M.H.U., Tufail M.A., Chen X.Y., Yan W.D. (2021). Intercropping of Peanut-Tea Enhances Soil Enzymatic Activity and Soil Nutrient Status at Different Soil Profiles in Subtropical Southern China. Plants.

[38] Xiao X.M., Cheng Z.H., Lv J., Xie J.M., Ma N., Yu J.H. (2019). A green garlic (*Allium sativum* L.) based intercropping system reduces the strain of continuous monocropping in cucumber (*Cucumis sativus* L.) by adjusting the micro-ecological environment of soil. PeerJ.

[39] Medeiros-Silva M., Franck W.L., Borba M.P., Pizzato S.B., Strodtman K.N., Emerich D.W., Stacey G., Poacco J.C., Carlini C.R. (2014). Soybean Ureases, but Not That of *Bradyrhizobium japonicum*, Are Involved in the Process of Soybean Root Nodulation. J. Agric. Food Chem..

[40] Cardone L., Castronuovo D., Perniola M., Cicco N., Candido V. (2019). Evaluation of corm origin and climatic conditions on saffron (*Crocus sativus* L.) yield and quality. J. Sci. Food Agric..

[41] Yuan B., Yu D., Hu A., Wang Y., Sun Y., Li C. (2023). Effects of green manure intercropping on soil nutrient content and bacterial community structure in litchi orchards in China. Front. Environ. Sci..

[42] Jing H., Wang H., Wang G., Liu G., Cheng Y. (2023). The mechanism effects of root exudate on microbial community of rhizosphere soil of tree, shrub, and grass in forest ecosystem under N deposition. ISME Commun..

[43] Kong H.G., Sang M.K., An J.H., Kim S., Jin Y.J., Song J. (2022). Changes in the Composition and Microbial Community of the Pepper Rhizosphere in Field with Bacterial Wilt Disease. Plant Pathol. J..

[44] Chauhan A., Guleria S., Balgir P.P., Walia A., Mahajan R., Mehta P., Shirkot C.K. (2017). Tricalcium phosphate solubilization and nitrogen fixation by newly isolated *Aneurinibacillus aneurinilyticus* CKMV1 from rhizosphere of *Valeriana jatamansi* and its growth promotional effect. Braz. J. Microbiol..

[45] Naureen Z., Rehman N.U., Hussain H., Gilani S.A., Khan A.L., Harrasi A.A. (2017). Exploring the potentials *of Lysinibacillus sphaericus* ZA9 for plant growth promotion and biocontrol activities against phytopathogenic fungi. Front. Microbiol..

[46] Eichorst S.A., Kuske C.R., Schmidt T.M. (2011). Influence of plant polymers on the distribution and cultivation of bacteria in the phylum Acidobacteria. Appl. Environ. Microbiol..

[47] Shiraishi A., Matsushita N., Hougetsu T. (2010). Nodulation in black locust by the Gammaproteobacteria *Pseudomonas* sp. and the Betaproteobacteria *Burkholderia* sp.. Syst. Appl. Microbiol..

[48] Schmitz E.V., Just C.L., Schilling K., Streeter M., Mattes T.E. (2023). Reconnaissance of Oxygenic Denitrifiers in Agriculturally Impacted Soils. mSphere.

[49] Viso N.P., Ortiz J., Maury M., Frene J.P., Iocoli G.A., Lorenzon C., Rivarola M., García F.O., Gudelj V., Faggioli V.S. (2024). Long-term maintenance rate fertilisation increases soil bacterial-archaeal community diversity in the subsoil and N-cycling potentials in a humid crop season. Appl. Soil Ecol..

[50] Chen F., Yu G., Sun Y.B., Zhang H.L., Tian X., Xia B. (2022). Characteristics of Soil Microbial Community Structure and Environmental Driving Factors in Farmland around Mercury Mining Areas. Environ. Sci..

[51] Jiang Y., Lin D., Guan X., Wang J., Cao G., Zhu D., Peng C. (2017). Effect of herbicide used with years (8 + 1) on soil enzymic activity and microbial population diversity. J. Soils Sediments.

[52] Gao J., Pei H., Xie H. (2021). Influence of allyl isothiocyanate on the soil microbial community structure and composition during pepper cultivation. J. Microbiol. Biotechnol..

[53] Li Y.Y., Feng J., Zheng L., Huang J.B., Yang Y., Li X.H. (2020). Intercropping with marigold promotes soil health and microbial structure to assist in mitigating tobacco bacterial wilt. J. Plant Pathol..

[54] Yan B., Li J.S., Xiao N.W., Qi Y., Fu G., Liu G.H., Qiao M.P. (2016). Urban-development-induced Changes in the Diversity and Composition of the Soil Bacterial Community in Beijing. Sci. Rep..

[55] Lopes L.D., Hao J.J., Schachtman D.P. (2021). Alkaline soil pH affects bulk soil, rhizosphere and root endosphere microbiomes of plants growing in a Sandhills ecosystem. FEMS Microbiol. Ecol..

[56] Shen C., Ni Y., Liang W., Wang J., Chu H. (2015). Distinct soil bacterial communities along a small-scale elevational gradient in alpine tundra. Front. Microbiol..

[57] Amtmann A., Troufflard S., Armengaud P. (2008). The effect of potassium nutrition on pest and disease resistance in plants. Physiol. Plant..

[58] Vuong T.M.D., Zeng J.Y., Man X.L. (2020). Soil fungal and bacterial communities in southern boreal forests of the Greater Khingan Mountains and their relationship with soil properties. Sci. Rep..

[59] Wan P., Peng H., Ji X.L., Chen X.L., Zhou H.M. (2021). Effect of stand age on soil microbial communities of a plantation *Ormosia hosiei* forest in southern China. Ecol. Inform..

