# Supplementary material for: Impact of Intercropping Five Medicinal Plants on Soil Nutrients, Enzyme Activity, and Microbial Community Structure in Camellia oleifera Plantations
Source: Microorganisms. 2024 Aug 8;12(8):1616. doi: 10.3390/microorganisms12081616 (PMC11356553; doi:10.3390/microorganisms12081616)
Supplement: Supplementary file 1 [file microorganisms-12-01616-s001.zip › microorganisms-3104259-supplementary.pdf]

Supplementary Materials

Table S1. Soil physical properties

Samples	SBD (g/cm3)	SMWC (%)	GC(%)	CW(%)	CP(%)	NCP(%)	SMC(%)	SWC(%)	FC(%)	WDC(%)
CK	1.28	19.99	31.21	28.57	3.65	0.33	16.65	15.67	28.31	2.91
EZ	1.22	16.03	30.11	27.46	3.34	0.31	13.8	13.21	26.94	3.17
JH	1.4	16.53	28.25	25.39	3.55	0.4	14.18	11.86	25.06	3.19
HDK	1.14	17.65	34.77	28.37	3.22	0.72	14.99	15.68	27.48	7.28
YDC	1.27	15.82	27.47	25.87	3.28	0.2	13.63	12.59	25.57	1.9
WZMT	1.2	17.58	34.07	27.88	3.35	0.73	14.91	14.7	27.05	7.02
ALL	1.25	17.27	30.98	27.26	3.4	0.45	14.69	13.95	26.74	4.25

Abbreviations: SBD-Soil bulk density; SWMC-soil mass water content, GC-maximum water holding capacity,CW-capillary water holding capacity, CP-capillary porosity,NCP-non-capillary porosity, SMC-soil water content, SWC-saturated water content, FC-field water holding capacity,WDC-water drainage capacity. EZZ: C.zed.-cover roots, EZK: C.zed.-C.ole. soil, EZY: C.ole.-cover roots. EZ: C. zedoaria/C. oleifera, JH: C. longa/C. oleifera, YDC: C. nutans/C. oleifera, HDK: F. Galangae/C. oleifera, WZMT: F. simplicissima/C. oleifera.
